# Stability of Reference Genes for Messenger RNA Quantification by Real-Time PCR in Mouse Dextran Sodium Sulfate Experimental Colitis

**DOI:** 10.1371/journal.pone.0156289

**Published:** 2016-05-31

**Authors:** Nour Eissa, Hayam Hussein, Hongxing Wang, Mohammad F. Rabbi, Charles N. Bernstein, Jean-Eric Ghia

**Affiliations:** 1 Immunology, University of Manitoba, Winnipeg, MB, Canada; 2 Children's Hospital Research Institute of Manitoba, University of Manitoba, Winnipeg, MB, Canada; 3 Department of Veterinary Clinical Sciences, College of Veterinary Medicine, Ohio State University, Columbus, Ohio, United States of America; 4 Internal Medicine section of Gastroenterology, University of Manitoba, Winnipeg, MB, Canada; 5 IBD Clinical and Research Centre, University of Manitoba, Winnipeg, MB, Canada; 6 Xuanwu Hospital, Capital Medical University, Beijing, China; Child & Family Research Institute, CANADA

## Abstract

**Background:**

Many animal models have been developed to characterize the complexity of colonic inflammation. In dextran sodium sulfate (DSS) experimental colitis in mice the choice of reference genes is critical for accurate quantification of target genes using quantitative real time PCR (RT-qPCR). No studies have addressed the performance of reference genes in mice DSS-experimental colitis. This study aimed to determine the stability of reference genes expression (RGE) in DSS-experimental murine colitis.

**Methods:**

Colitis was induced in male C57BL/6 mice using DSS5% for 5 days, control group received water. RNA was extracted from inflamed and non-inflamed colon. Using RT-qPCR, comparative analysis of 13 RGE was performed according to predefined criteria and relative colonic *TNF-α* and *IL-1β* gene expression was determined by calculating the difference in the threshold cycle.

**Results:**

Colitis significantly altered the stability of mucosal RGE. Commonly used glyceraldehyde-3-phosphate dehydrogenase (*Gapdh*), β-actin (*Actb*), or β2-microglobulin (*β2m)* showed the highest variability within the inflamed and control groups. Conversely, TATA-box-binding protein (*Tbp*) and eukaryotic translation elongation factor 2 (*Eef2*) were not affected by inflammation and were the most stable genes. Normalization of colonic *TNF-α* and *IL-1β* mRNA levels was dependent on the reference gene used. Depending on the genes used to normalize the data, statistical significance varied from significant when *TBP / Eef2* were used to non-significant when *Gapdh*, *Actb* or *β2m* were used.

**Conclusions:**

This study highlights the appropriate choice of RGE to ensure adequate normalization of RT-qPCR data when using this model. Suboptimal RGE may explain controversial results from published studies. We recommend using *Tbp* and *Eef2* instead of *Gapdh*, *Actb* or *β2m* as reference genes.

## Background

Inflammatory bowel disease (IBD) is a group of disorders where segments of the gastrointestinal tract become inflamed and ulcerated. Ulcerative colitis (UC) is one form of IBD. UC is an idiopathic chronic relapsing–remitting inflammatory disorder that affects the colon and is characterized by diarrhea and rectal bleeding. The etiology and pathogenesis of UC are still unknown, and accumulating evidence has indicated that sustained intestinal infection, commensal microbiota, mucosal barrier defects, mucosal immune dysregulation, genetic, environmental factors and stress related factors are involved in UC pathophysiology. UC is globally distributed, and the prevalence in Europe and North America is 24.3 and 19.2 per 100,000 individuals, respectively, and 6.3 per 100,000 individuals in Asia and the Middle East [1]. The growing prevalence of this disease impacts on the economic and health care burdens that affect patients’ quality of life. Treatment for a UC patient in the United States costs approximately $15,020 annually [2].

Over the last two decades, many animal models have been developed to characterize the complexity of UC pathophysiology. These models have helped to study molecular mechanisms and to determine potential human therapeutics [3]. The most widely used mouse model of colonic injury/repair is dextran sodium sulfate (DSS), a water-soluble negatively charged chemical with anticoagulant properties. DSS causes chemical injury in the intestinal mucosa and results in exposure of the lamina propria (LP) and sub-mucosal compartment to luminal antigens; enteric bacteria then promote the development of inflammation. Although the DSS-model is commonly used in UC research, controversy exists regarding the nature of the injury because some experts have classified this model as an injury/repair model and not as UC-model. However, given its ease of administration (in drinking water), the simplicity of controlling the dosage (severity), interval (recovery process) and its cost effectiveness [4], the DSS model is one of the most widely used models of colonic inflammation (over 4000 entries in PubMed).

Quantitative real-time polymerase chain reaction (RT-qPCR) is a powerful tool for gene expression studies and it is characterized by high sensitivity, specificity, wide quantification range for high throughput and accurate expression profiling of selected targets [5]. The most common method for data normalization includes the use of endogenous normalizer genes, known as internal control, housekeeping or reference genes, which are required for RT-qPCR data analysis using the comparative cycle threshold (Ct) method [6]. Therefore, the best endogenous normalizer gene should be characterized by consistent mRNA expression in all samples under study, irrespective of tissue type, disease state or experimental conditions, and it should have expression levels comparable to that of the target gene [7].

Although an ideal endogenous normalizer gene has not yet been discovered, many different genes have been selected and used depending on the insult, lesion or disease conditions in humans, experimental conditions, cell culture or experimental animal models [8, 9]. Several mathematical methods have been established to analyze the variability of candidate endogenous normalizer genes such as geNorm [10], bestKeeper [11], normFinder [12] and the comparative delta Ct method [13]. Most of these methods use a stability value, a measure of variation observed within the samples, to rank each gene, where a lower stability value refers to low variation and, therefore, correctness as a reference gene compared to other candidates. Thus, careful selection, evaluation and validation of the endogenous normalizer gene is crucial for data interpretation and is necessary to avoid possible data inaccuracies from the use of an inappropriate endogenous normalizer gene [14, 15].

To our knowledge, no validated endogenous normalizer genes related to the DSS-experimental model have been reported for relative mRNA quantification. Here, we examine the problems associated with using non-validated or unsuitable endogenous normalizer genes and their impact on data accuracy. Therefore, the aim of this study was to confirm the validity of the following 13 potential endogenous normalizer genes: *Gapdh*, *Actb*, *β2m*, *Hmbs*, *Hprt*, *RpLp0*, *Tbp*, *Gusb*, *Ppia*, *Oaz1*, *Nono*, *Tfrc* and *Eef2*, which are were selected after reviewing the literature for studies that used a mouse DSS-model. This approach provided a detailed analysis of common and novel reference genes for the future use of the DSS-model in relation to RT-qPCR analyses. Moreover, using the 13 housekeeping genes related to the expression of two major colonic pro-inflammatory cytokines in inflamed and normal tissues, we aimed to find the most suitable endogenous normalizer genes for mRNA expression studies using DSS-induced colitis in mice.

## Materials and Methods

### Ethics statement

Mice were cared for and used in accordance with the Guide of Canadian Council on Animal Care in science (CCAC) for the Care and Use of Laboratory Animals for Scientific Purposes. All experiments were carried out in strict accordance with the recommendations approved by the University of Manitoba Animal Ethics Committee (Protocol # 15–010) and conducted under the Canadian guidelines for animal research.

### Animals & DSS-model

Male C57BL/6 (6–8 weeks old) mice were purchased from Charles Rivers (Sherbrook, Canada) and maintained in the animal care facility at the University of Manitoba under specific pathogen-free conditions. DSS (molecular weight [MW], 40 kDa; MP Biomedicals, Soho, OH) was added to the drinking water at a final concentration of 5% (wt/vol) for 5 days (n = 6) [16–19]. Controls (n = 6) were time-matched and consisted of mice that received normal drinking water only. The 5% DSS concentration and 5 days treatment is the optimal set-up for the sanitary condition of our facility. Mean DSS consumption was noted per cage each day.

### Assessment of colitis severity

To confirm the induction of colitis, we evaluated and quantified classical experimental inflammatory parameters. The disease activity index (DAI) was scored from day 0 to 5 during DSS treatment, and then mice were sacrificed for macroscopic scoring, as described previously [20]. Formalin-fixed colon segments isolated from the splenic flexure were paraffin-embedded, and 3-mm sections were stained using hematoxylin-eosin (H&E) (Sigma, Mississauga, Canada). Colonic damage was scored based on a published scoring system that considers architectural derangements, goblet cell depletion, edema/ulceration and degree of inflammatory cell infiltrate [20]. On the sacrifice day, blood was collected by intra-cardiac puncture in anaesthetized (Isoflurane, Abbott, Toronto, Canada) mice, and C-reactive protein (CRP) levels in the serum were determined using an ELISA commercial kit (Immuno- Consultants, Portland, OR, USA). Colonic myeloperoxidase (MPO) activity (Hycult Biotech, PA, USA) level was quantified using ELISA (R&D Systems, Inc., MN, USA). Colonic tumor necrosis factor-alpha (TNF-α), interleukin (IL)-6 and IL-1beta (β) protein levels were quantified using ELISA (R&D Systems, Inc., MN, USA).

### Colon RNA extraction and cDNA synthesis

Approximately 30–40 mg of colon tissue was used for total RNA extraction using TRIzol (Gibco BRL, Life Technologies, NY, USA), according to manufacturer’s instructions. Quality and quantity of RNA were determined by measuring the absorbance at 260 and 280 nm using NanoDrop ND-1000 UV-Vis Spectrophotometer (Thermo Fisher Scientific, Waltham, MA, USA). All samples had an absorption ratio A260/A280 greater than 1.8. RNA (1 μg) from each sample was treated with RQ1 RNase-Free DNase® (Promega Corporation, Madison, WI, USA), according to the manufacturer’s instructions, to remove genomic DNA contamination. Reverse transcription was performed using SuperScript VILO cDNA Synthesis Master Mix (Invitrogen, Grand Island, NY, USA), according to the manufacturer’s instructions, in an Eppendorf Thermo cycler at 25°C for 10 min, followed by 42°C for 60 min, and 85°C for 5 min. Samples were then cooled to 4°C. cDNA samples were stored at -20°C for RT-qPCR analysis.

### Primer design

Thirteen candidate reference genes were selected based on their common usage as endogenous control genes in previous studies. The candidate genes were *Gapdh*, *Actb*, *β2m*, *Hmbs*, *Hprt*, *RpLp0*, *Tbp*, *Gusb*, *Ppia*, *Oaz1*, *Nono*, *Tfrc and Eef2*. The primers were designed from nucleotide sequences identified using NCBI BLAST (http://blast.ncbi.nlm.nih.gov/Blast.cgi), according to the following properties: melting temperature (Tm) of 58–62°C, GC content of 45–55%, length 18–22 bp and amplicon size was specified to be between 75 and 175 bp. Primers were ordered from Life Technologies with their certificates of analysis. The primer characteristics of nominated reference genes are listed in Table 1.

**Table 1:** Description of selected candidate endogenous control and target genes.

Gene Symbol	Gene Name	Gene Function		Sequence 5' ->3'	Length	TM	Location	Amplicon Size	Gene Accession Number
***Gapdh***	Glyceraldehyde3-phosphate dehydrogenase	Glycolysis pathway enzyme	Forward	AGGTCGGTGTGAACGGATTTG	21	62.6	8–28	95	NM_008084
			Reverse	GGGGTCGTTGATGGCAACA	19	62.6	84–102		
***Actb***	Beta Actin	Cytoskeletal structural protein	Forward	GGCTGTATTCCCCTCCATCG	2	61.8	103–84	154	NM_007393
			Reverse	CCAGTTGGTAACAATGCCATGT	22	61.1	237–216		
***β2m***	Beta 2 Microglobulin	Beta-chain of major histocompatibility complex	Forward	TTCTGGTGCTTGTCTCACTGA	21	61	26–46	104	NM_009735
			Reverse	CAGTATGTTCGGCTTCCCATTC	22	61	129–108		
***Hmbs***	Hydroxymethylbilane synthase	Heme biosynthetic pathway enzyme	Forward	AAGGGCTTTTCTGAGGCACC	20	62.4	699–718	78	NM_001110251
			Reverse	AGTTGCCCATCTTTCATCACTG	22	60.6	776–755		
***Hprt***	Hypoxanthine phosphoribosyl transferase	Metabolic salvage of purines	Forward	TCAGTCAACGGGGGACATAAA	21	60.8	324–344	142	NM_013556
			Reverse	GGGGCTGTACTGCTTAACCAG	21	62.4	465–445		
***Rplp0***	Ribosomal Protein Large P0	Structural constituent of ribosome	Forward	AGATTCGGGATATGCTGTTGGC	22	62.5	290–311	109	NM_007475
			Reverse	TCGGGTCCTAGACCAGTGTTC	21	62.7	398–378		
***Tbp***	TATA-box-binding protein	General transcription factor	Forward	ACCGTGAATCTTGGCTGTAAAC	22	60.8	442–463	86	NM_013684
			Reverse	GCAGCAAATCGCTTGGGATTA	21	61.3	527–507		
***Gusb***	Glucuronidase, Beta	Lysosomal exoglycosidase	Forward	GGCTGGTGACCTACTGGATTT	21	61.5	722–742	131	NM_010368
			Reverse	GGCACTGGGAACCTGAAGT	19	61.5	852–834		
***Ppia***	Peptidylprolyl Isomerase A	Protein coding, a cyclosporin binding-protein	Forward	GAGCTGTTTGCAGACAAAGTTC	22	60.2	67–88	125	NM_008907
			Reverse	CCCTGGCACATGAATCCTGG	20	62.6	191–172		
***Oaz1***	Ornithine Decarboxylase Antizyme 1	Cell growth & Proliferation	Forward	CGCACCATGCCGCTTCTTA	19	63	94–112	169	NM_008753
			Reverse	ATCCCGCTGACTGTTCCCT	19	62.6	262–244		
***Nono***	Non-POU Domain Containing, Octamer-Binding	Transcriptional regulation and RNA splicing	Forward	AAAGCAGGCGAAGTTTTCATTC	22	60	301–322	77	NM_023144
			Reverse	ATTTCCGCTAGGGTTCGTGTT	21	61.7	377–357		
***Tfrc***	Transferrin Receptor	Endocytosis & HIF1 signalling pathway	Forward	GTTTCTGCCAGCCCCTTATTAT	22	60.1	1495–1516	152	NM_011638
			Reverse	GCAAGGAAAGGATATGCAGCA	21	60.7	1646–1626		
***Eef2***	Eukaryotic Translation Elongation Factor 2	Protein Synthesis	Forward	TGTCAGTCATCGCCCATGTG	20	62.2	65–84	123	NM_007907.2
			Reverse	CATCCTTGCGAGTGTCAGTGA	21	62.1	187–167		
***TNF-α***	Tumor Necrosis Factor Alpha	Pro-inflammatory cell signalling protein (Cytokine)	Forward	CCCTCACACTCAGATCATCTTCT	23	60.9	230–252	61	NM_013693
			Reverse	GCTACGACGTGGGCTACAG	19	62.1	290–272		
***IL-1β***	Interleukin (IL)-1 β	Pro-inflammatory cell signalling protein	Forward	GCAACTGTTCCTGAACTCAACT	22	60.7	4–25	89	NM_008361
			Reverse	ATCTTTTGGGGTCCGTCAACT	21	61.4	92–72		

### Quantitative real-time polymerase chain reaction

Real-time PCR (RT-PCR) reactions were performed in a Roch lightCycler 96 Real-Time System using Power SYBR green master mix (Life Technologies), according to the manufacturer’s instructions, in a final volume of 20 μl reactions. The PCR conditions were as follows: 95°C for 10 minutes, followed by 40 cycles at 95°C for 15 seconds and at 60°C for 60 seconds. To verify the specificity of each primer, a melting-curve analysis was included (65–95°C with fluorescence measured every 0.5°C). The absence of contamination from either genomic DNA amplification or primer dimers formation was ensured using two types of controls: the first without reverse transcriptase (no-RT control, one for each RNA) and the second with no DNA template (NTC control, one for each primer pair). All RT-qPCRs were run in duplicate, the average standard deviation within duplicates of all samples studied was 0.25 cycles.

### PCR primer efficiency

RT-qPCR efficiencies in the exponential phase were calculated for each primer pair using standard curves (5-point 5-fold serial dilution of pooled cDNA that included equal amounts from the samples set), the mean Ct values for each serial dilution were plotted against the logarithm of the cDNA dilution factor and calculated according to the equation E = 10^[-1/slope]^ [21], where the slope is the gradient of the linear regression line. The linear dynamic range was determined by the standard curve and correlation coefficients (R^2^) for each gene as reported.

### Analysis of gene stability

To rank the stability of the selected internal control genes, publicly-available tools (geNorm, BestKeeper, Comprehensive Ranking) were used. geNorm ranked the reference genes based on their M value; a lower value of the M average expression stability represented stable expression whereas a high value represented less stable expression [10]. BestKeeper calculated the gene expression stability for candidate genes based on each candidate gene’s Ct values [11]. Finally, Comprehensive Ranking [22], which is a web-based comprehensive integrated database, applied a methodology to compare candidate reference gene performance to compensate weaknesses of the individual tools such as geNorm and BestKeeper. Comprehensive Ranking, based on the ranking from each tool, gave an appropriate weight to each reference gene and calculated the geometric mean of their weights for the overall final ranking. Raw Ct values (untransformed data) were used directly for data importing of integrated database. These tools take into consideration several parameters, including Ct standard deviation (SD) for respective cDNA detection within different samples.

### Analysis of appropriateness of selected reference genes in DSS-experimental colitis using specific targeted genes

Two genes were selected to test the suitability of the 13 reference genes, and to highlight the significance of choosing an optimal reference gene to quantify target gene mRNA expression levels. TNF-α and IL-1β were selected based on their contribution to the development of experimental colitis. Up-regulation of these cytokines in inflamed colon has been extensively reported and they are also considered to be classical pro-inflammatory mediators in UC. Therefore, using the 13 reference genes, the relative *TNF-α* and *IL-1β* transcript expression in control and DSS groups was determined by calculating differences in the comparative threshold cycle (ΔΔCt) and also, the difference in threshold cycle (ΔCt) methods [6].

### Selected reference genes stability in DSS-experimental colitis using External RNA Controls Consortium (ERCC)

External RNA Controls Consortium (ERCC) RNA Spike-In Mixes (Life Technologies, USA) and RNAs from control and DSS groups were used according to the manufacture instructions. Reverse transcription was performed using SuperScript VILO cDNA Synthesis Master Mix (Invitrogen, Grand Island, NY, USA). To control for template quality, the threshold cycle (Ct) values of 3 selected control ERCC RNAs (ERCC-0002, ERCC-00113, ERCC-0074) were concurrently assessed, and only experimental samples that showed threshold cycles within 2 cycles of the median values of ERCC RNA were engaged in the relative quantification of the expression level of the reference genes. The expression values for the reference genes were calculated by normalizing their expression to the Ct values for ERCC-00113, one of the choice of the control ERCC RNAs, using the ΔΔCt method [6].

### Statistical analysis

The results were compared and analyzed using a one-way/two-way analysis of variance (ANOVA) and the Student’s *t*-test. Differences were reported as statistically significant when P < 0.05. GraphPad Prism 6 (GraphPad Software, Inc. La Jolla, CA, USA) was used for statistical procedures and graph plotting.

## Results

### Colitis induction

**Fig 1:**
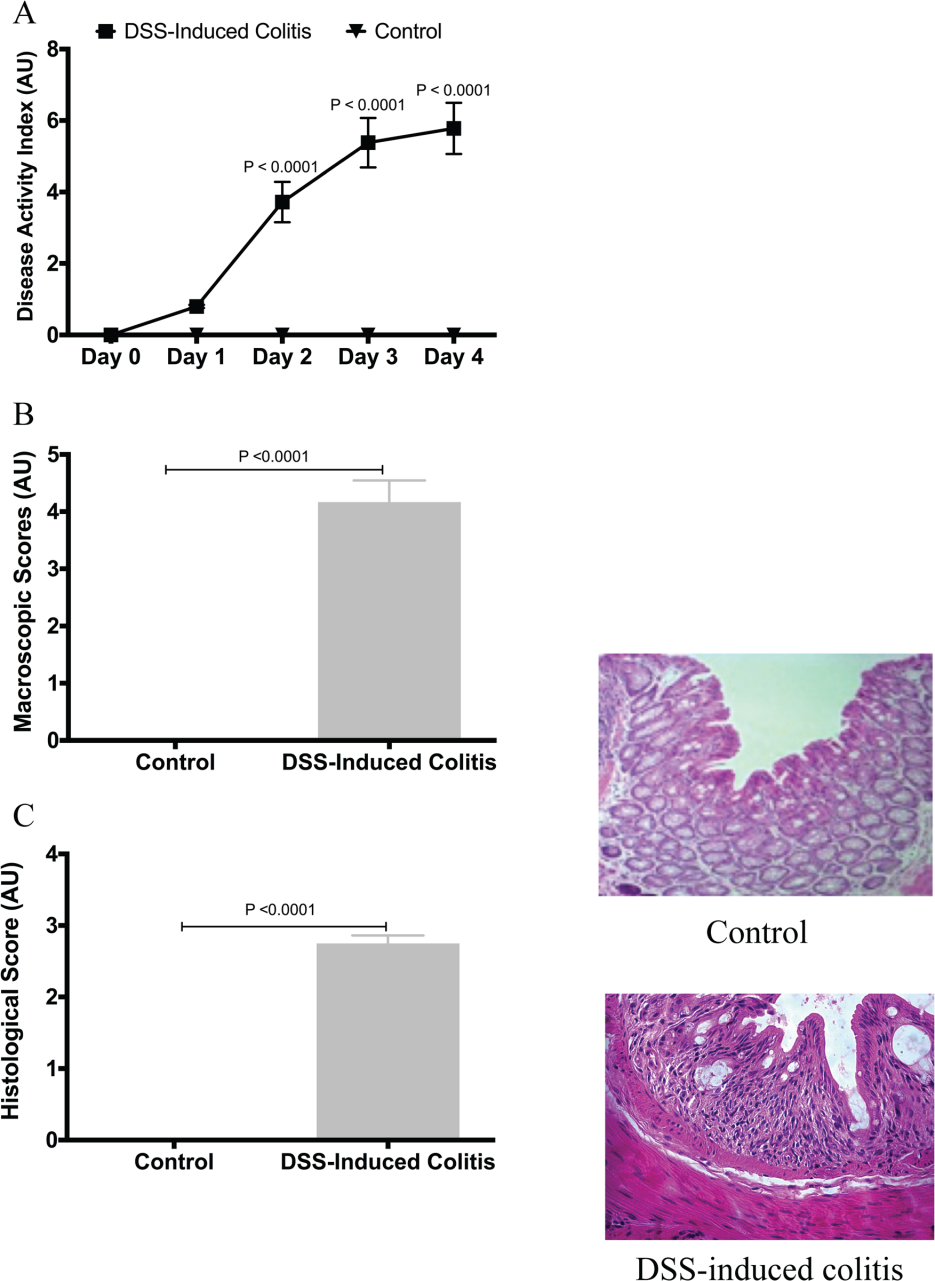
Confirmation of colitis induction by dextran sulphate sodium (DSS). (A) Disease activity index, repeated measures ANOVA analysis followed by the multiple comparisons post hoc analysis; (B) macroscopic scores; (C) histological score. Control represents data obtained in non-colitic non-treated mice (n = 6/group). Student’s *t*-test analyses were used to compare DSS-treated group to the control group. Data are presented as the mean ± SD.

**Fig 2:**
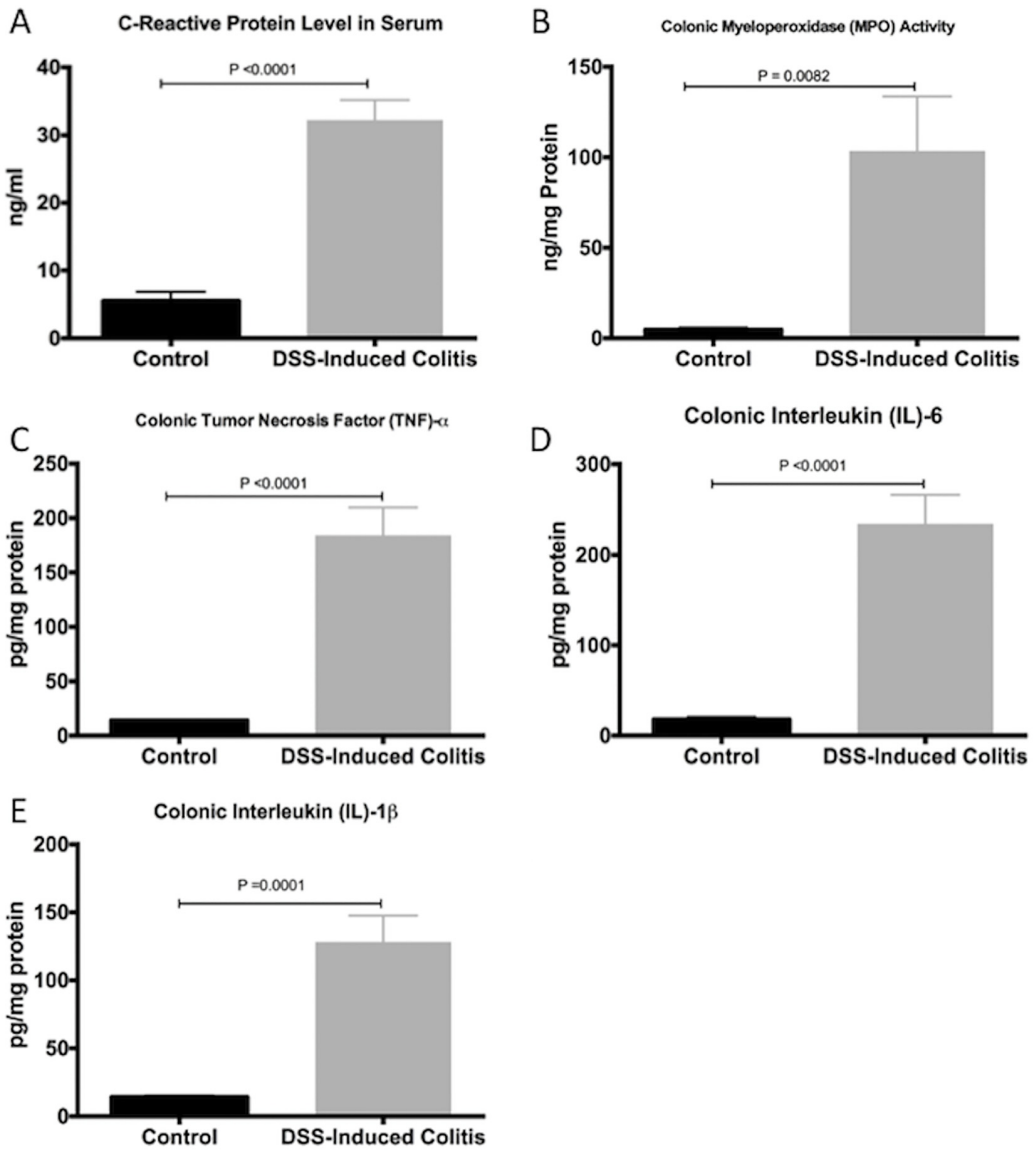
Systematic confirmation of colitis induction by dextran sulphate sodium (DSS). (A) C-reactive protein serum level; (B) myeloperoxidase (*MPO*) activity in the colon; and colonic pro-inflammatory mediators: (C) *TNF-α*, (D) *IL-6* and (E) *IL-1β*. Control represents data obtained in non-colitic non-treated mice (n = 6/group). Student’s *t*-test analyses were used to compare DSS-treated group to the control group. Data are presented as the mean ± SD.

We first confirmed the induction and development of experimental colitis in the DSS-treated group compared to control. When compared to the control group, the DSS-treated group showed more disease onset and greater severity, based on the DAI, and on macroscopic and histological scores (Fig 1A, 1B and 1C). This was confirmed by a 6- and 20-fold increase in serum CRP levels and colonic MPO activity (Fig 2A and 2B), respectively. The protein levels of colonic pro-inflammatory cytokines (TNF-α, IL-6, IL-1β) were also increased by 13-, 15- and 8-fold, respectively (Fig 2C, 2D and 2E).

### Specificity and primer efficiency

RT-qPCR was used to determine the performance for each primer set via primer specificity and efficiency. The dissociation curve following RT-qPCR confirmed the amplicon specificity. A single peak in the melting curve analyses for each of the 13 sets of primers referred to high specificity. The amplification efficiency for all primer sets ranged from 87% to 120% and the correlation coefficients (R^2^) were greater than 0.98 (S1 Fig).

### Reference gene expression profiles

**Fig 3:**
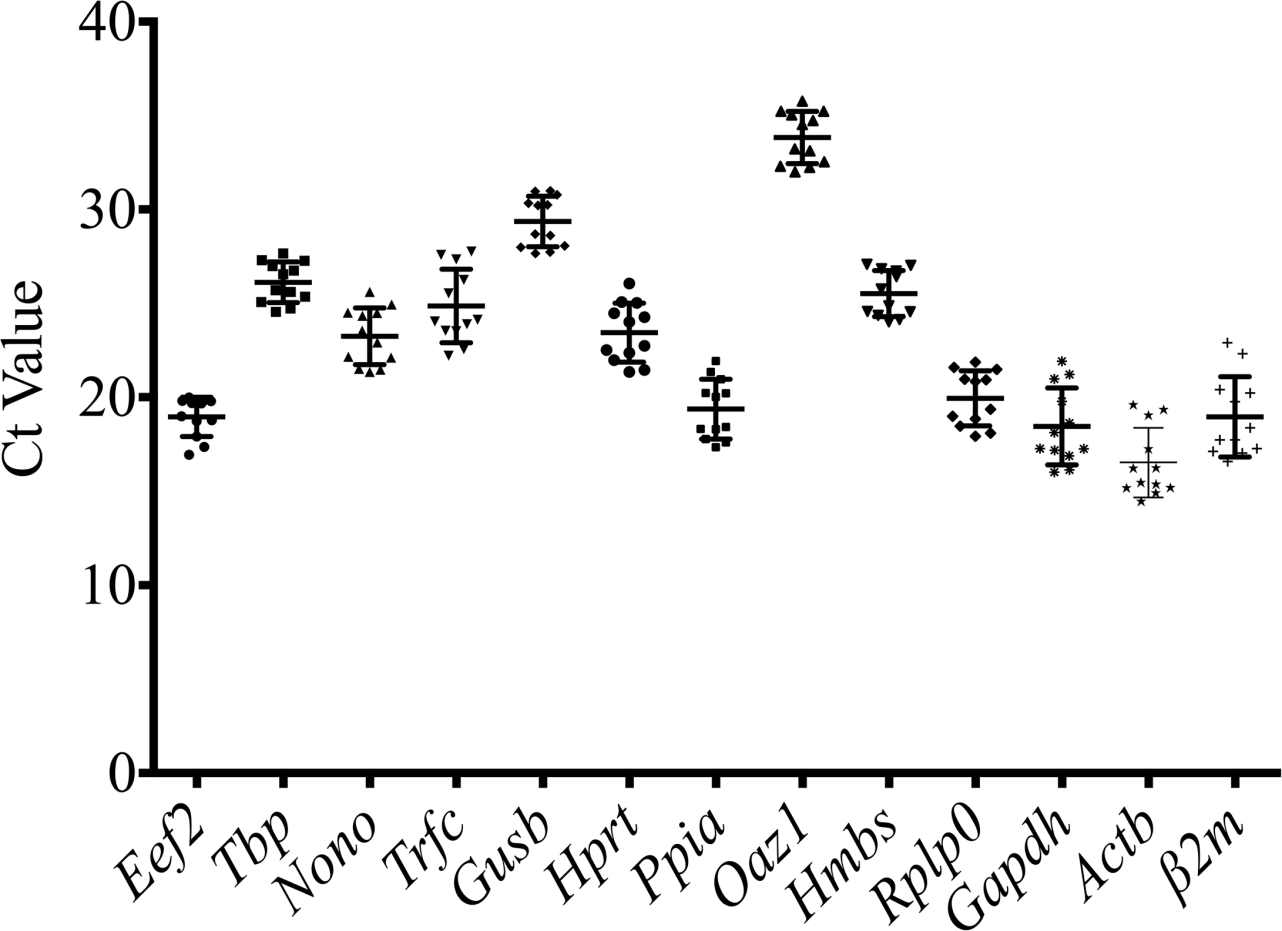
Range of quantification/threshold cycle (Ct) values of the candidate reference genes. A scatter dot plot displays the mean value and standard deviation in all colon samples (n = 12).

The expression levels of the 13 reference genes were determined using Ct and descriptive statistics (mean, SD, median, min, max) (Fig 3, Table 2) in all tested samples. The mean Ct values for reference genes ranged between 15 and 32, with most between 18 and 25. *Oaz1* had the highest median Ct value (33.88), which indicated a relatively low expression at the colon level, while *Actb* had the lowest median Ct value (Ct = 15.85), which indicated relatively high expression at the colon level. The variable transcript abundance of the 13 reference genes demonstrated different expression levels in the same experimental set. Taking into consideration all the samples, the lowest standard deviations were determined for *Eef2* (SD = 1.05) and *Tbp* (SD = 1.084), defining those two reference genes as having the lowest variability, while *Gapdh* (SD = 2.05), *Trfc* (SD = 1.963), *β2m* (SD = 2.137) and *Actb* (SD = 1.842) were defined as having the highest fluctuations in their mRNA expression.

**Table 2:** Descriptive performance of candidate reference genes.

Reference Gene	Minimum	Maximum	Median	Mean	Standard Deviation	Efficiency %	R^2^
*Eef2*	16.94	19.98	19.33	18.95	1.05	101	1.00
*Tbp*	24.55	27.65	26.12	26.12	1.084	120	0.98
*Nono*	21.34	25.61	23.23	23.24	1.515	99	1.00
***Trfc***	22.21	27.76	24.08	24.86	**1.963**	99	0.99
*Gusb*	27.65	30.99	29.46	29.36	1.343	94	0.99
*Hprt*	21.34	26.06	23.37	23.44	1.571	105	0.99
*Ppia*	17.34	21.93	19.22	19.37	1.593	99	1.00
*Oaz1*	32.01	35.78	33.88	33.84	1.388	96	0.99
*Hmbs*	24.01	27.08	25.31	25.52	1.228	104	1.00
*Rplp0*	17.92	21.87	20.1	19.94	1.469	105	1.00
***Gapdh***	16.01	21.92	17.69	18.44	**2.05**	93	0.99
***Actb***	14.46	19.6	15.85	16.52	**1.842**	91	0.99
***β2m***	16.58	22.9	18.05	18.95	**2.137**	87	1.00

### Effect of inflammation on candidate reference gene mRNA expression in colonic specimens

**Fig 4:**
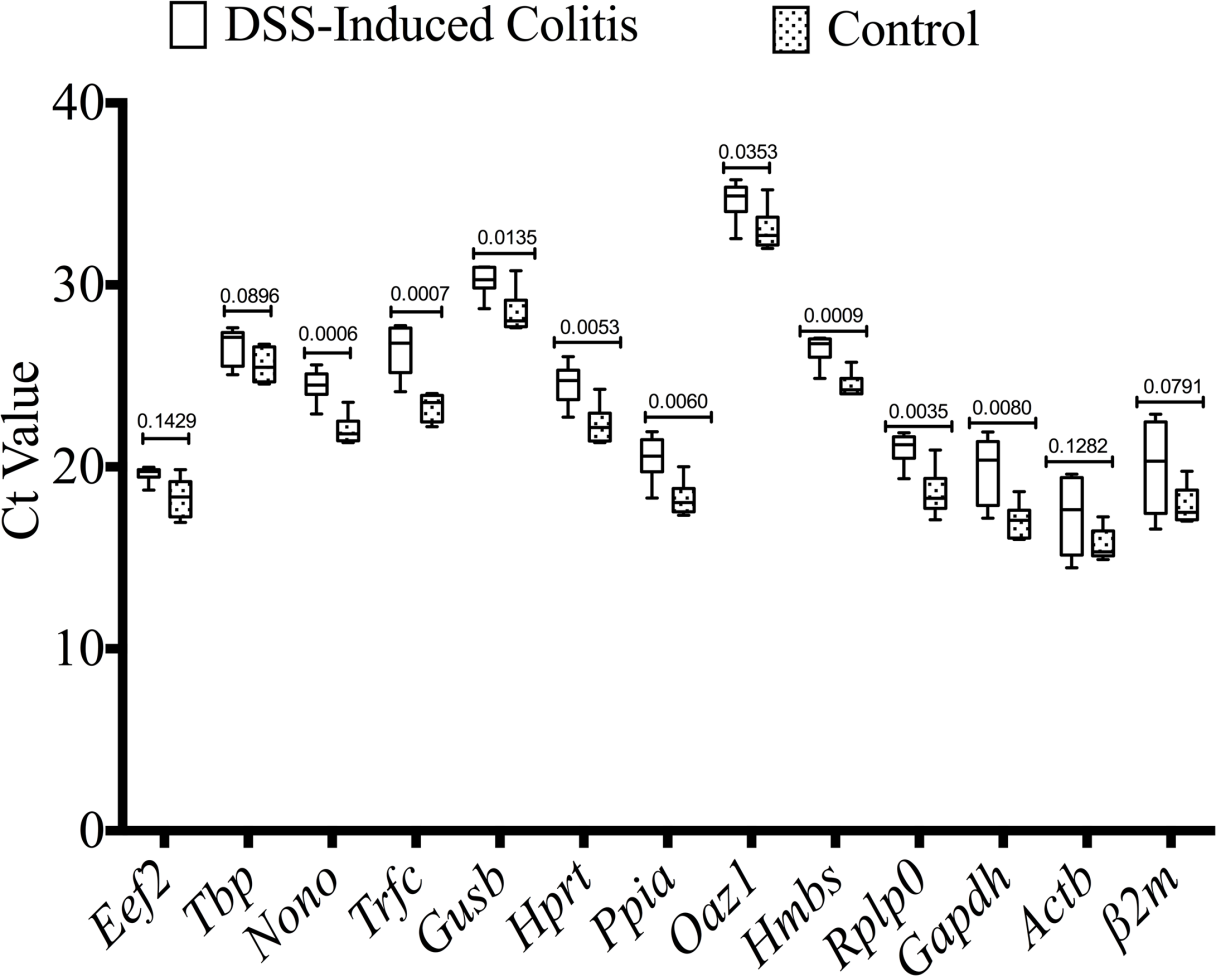
Effect of inflammation on candidate reference gene expression in colonic specimens. DSS-induced colitic and non-inflamed control colon groups are defined in the Materials and Methods. Several genes were found to show altered expression between samples from control and DSS-induced colitis group (n = 6/group). Student’s t-test was used for comparison between the groups. Ct, threshold cycle.

To examine whether the development of inflammation had any effect on mRNA expression levels, the candidate gene mRNA from the DSS group compared to the non-colitic control group were determined. As described in Fig 4, mRNA expression levels of several reference genes, such as *Nono* (P = 0.0006), *Trfc* (P = 0.0007), *Gusb* (P = 0.0135), *Hprt* (P = 0.0053), *Ppia* (P = 0.0060), *Hmbs* (P = 0.0009), *Rplp0* (P = 0.0035) and *Gapdh* (P = 0.0080) were significantly altered under inflammatory conditions. Non-significant differences with justified data distribution were reported for *Tbp* and *Eef2*. Although there is no significant alteration between colitic and non-colitic groups for *β2m and Actb*, a high variability was shown by the high standard deviations (2.137 and 1.842, respectively). *Oaz1* expression demonstrated a mean Ct value of 33.84, high standard deviation (1.338) and was significantly altered under inflammatory conditions (P = 0.0335).

### Stability of reference genes

To assess and rank reference gene stability, three different tools were used: geNorm gene, BestKeeper gene stability and final comprehensive gene stability ranking.

#### geNorm gene stability

**Fig 5:**
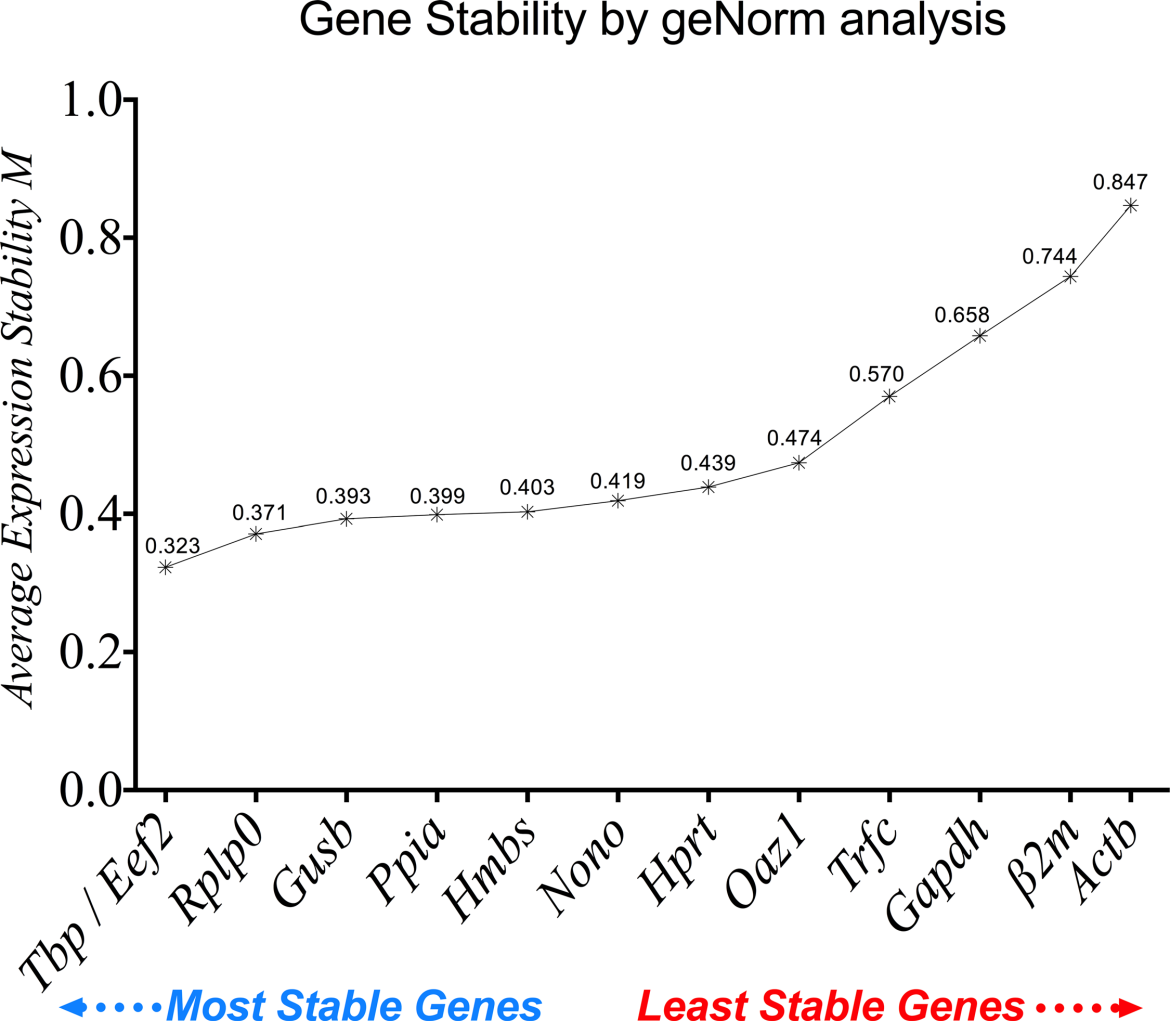
The average expression stability values of the 13 reference genes analyzed by geNorm. A lower M value refers to higher gene expression stability.

The average gene expression stability (M value) of the 13 reference genes tested was calculated using geNorm via an integrated web database platform [22]. All selected reference genes showed expression stability, with M < 1.5 (the default cutoff M value was determined by geNorm). The most stable reference genes were *Tbp/Eef2* followed by *Rplp0* and *Gusb*, while *Gapdh*, *β2m* and *Actb* exhibited the lowest gene expression stability (Fig 5). The order of the 13 tested reference genes, from the most stable to the least stable was: *Tbp* (0.323), *Eef2* (0.323), *Rplp0* (0.371), *Gusb* (0.393), *Ppia* (0.399), *Hmbs* (0.403), *Nono* (0.419), *Hprt* (0.439), *Oaz1* (0.474), *Trfc* (0.570), *Gapdh* (0.658), *β2m* (0.744) and *Actb* (0.847).

#### BestKeeper gene stability

**Fig 6:**
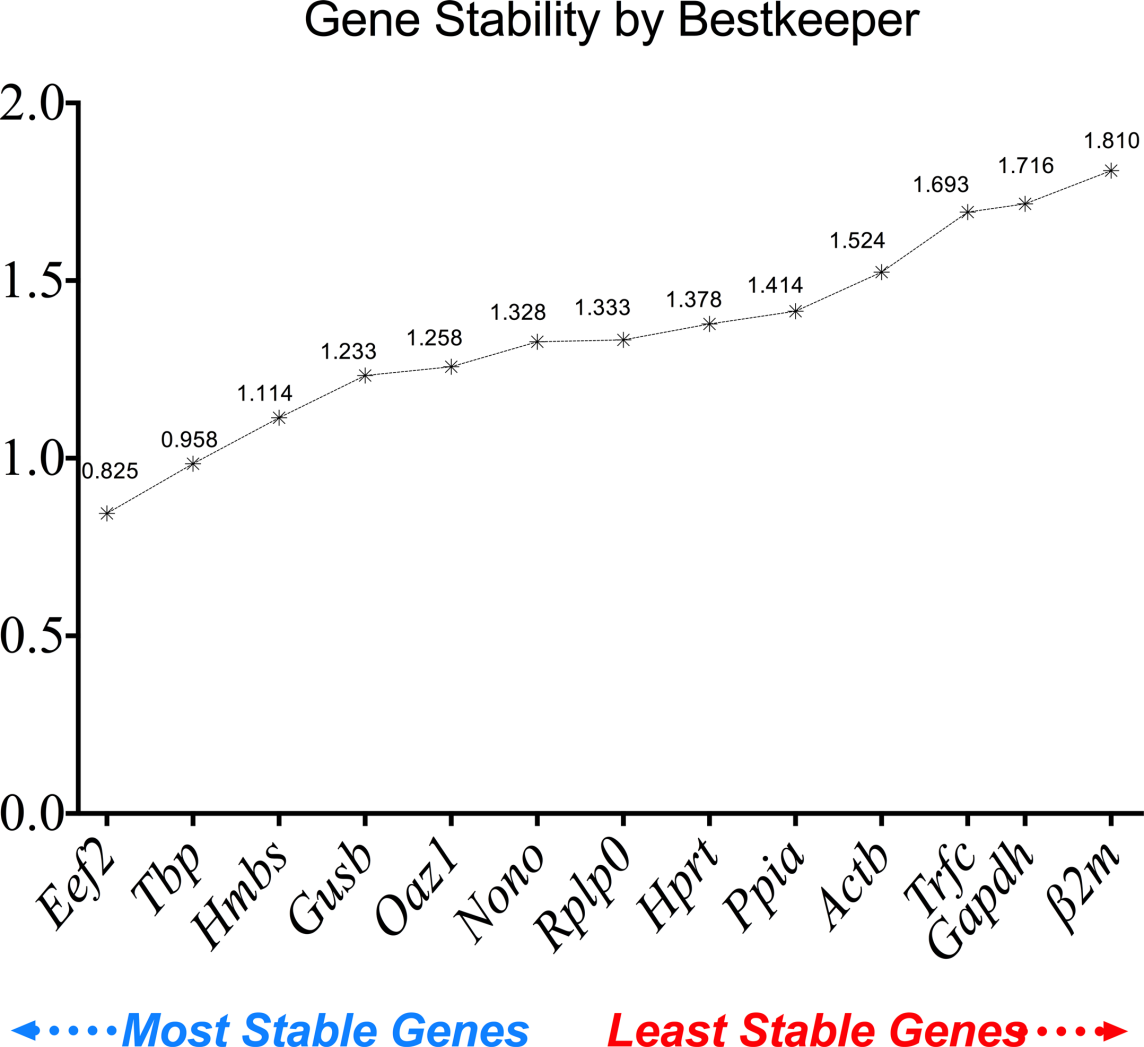
Reference gene stability performance of the 13 reference genes analyzed using BestKeeper. Lower values refer to higher stability and higher values refer to a lower stability.

The Bestkeeper gene stability was also calculated. From most stable to least stable, the ranking is as follows: *Eef2* (0.825), *Tbp* (0.958), *Hmbs* (1.114), *Gusb* (1.233), *Oaz1* (1.258), *Nono* (1.328), *Rplp0* (1.333), *Hprt* (1.378), *Ppia* (1.414), *Actb* (1.524), *Trfc* (1.693), *Gapdh* (1.716) and *β2m* (1.810) (Fig 6). BestKeeper gene stability expression showed the same pattern of results for the two most stable and the three least stable reference genes when compared with the geNorm analysis.

#### Final comprehensive gene stability ranking

**Fig 7:**
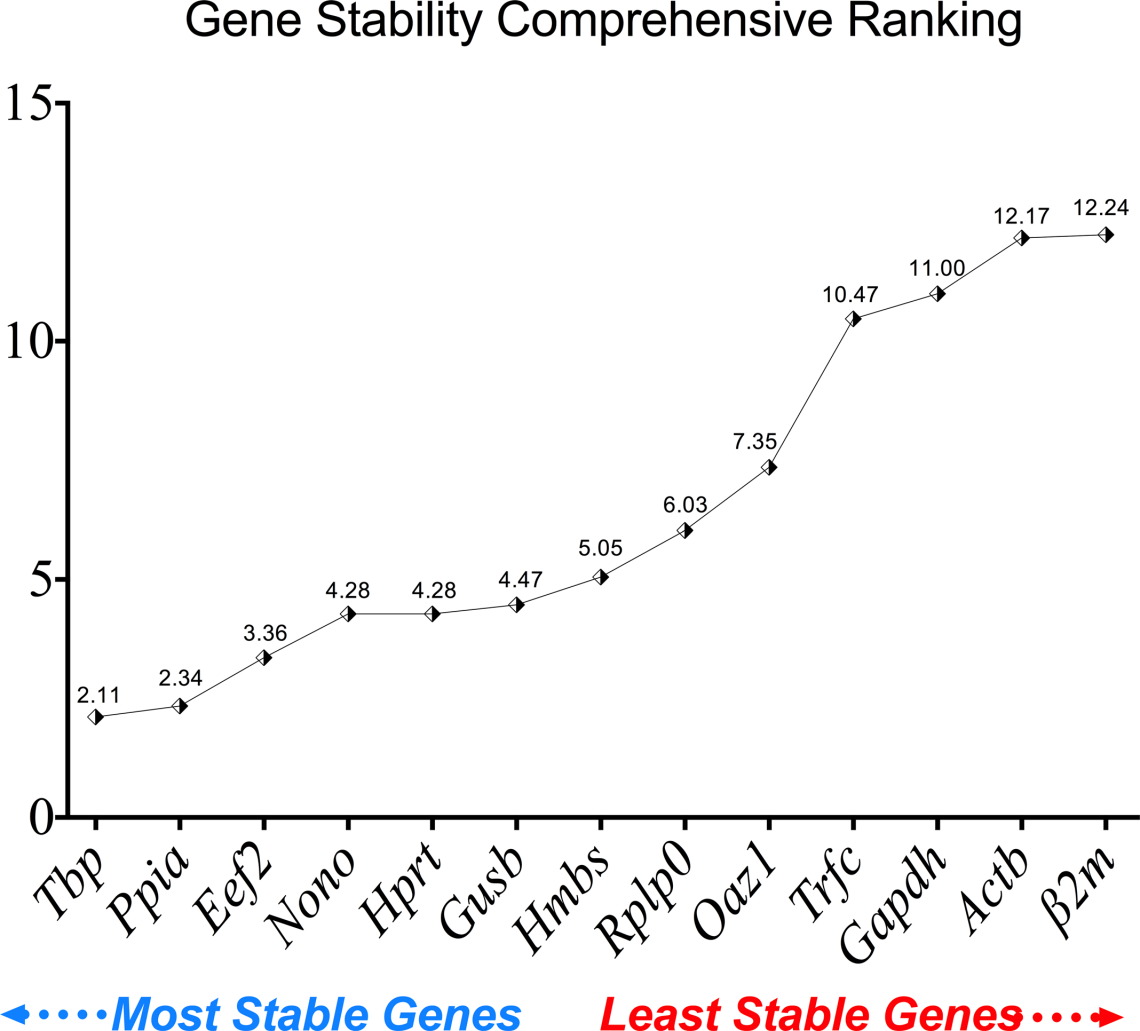
Comprehensive gene stability ranking for the 13 reference genes used in DSS-induced colitis and control groups. RT-qPCR was performed for each of the 13 reference genes using the same RNA samples from control and DSS-induced colitis groups (inflamed and non-inflamed areas).

Reference gene stability expression was also calculated using the comprehensive ranking. As shown in Fig 7, the stability rank from the most stable to the least stable was as follows: *Tbp* (2.11), *Ppia* (2.34), *Eef2* (3.36), *Nono* (4.28), *Hprt* (4.28), *Gusb* (4.47), *Hmbs* (5.05), *Rplp0* (6.03), *Oaz1* (7.35), *Trfc* (10.47), *Gapdh* (11.00), *Actb* (12.17) and *Β2M* (12.24). Data from the three tools showed that *Gapdh*, *Actb* and *β2m* are suboptimal candidates for normalization of target gene expression in the DSS-model, because their stability was affected by the presence of inflammation and the experimental conditions. Conversely, *Tbp*, *Ppia* and *Eef2* showed consistent expression stability.

### Effect of reference gene selection on the target mRNA relative expression

**Fig 8:**
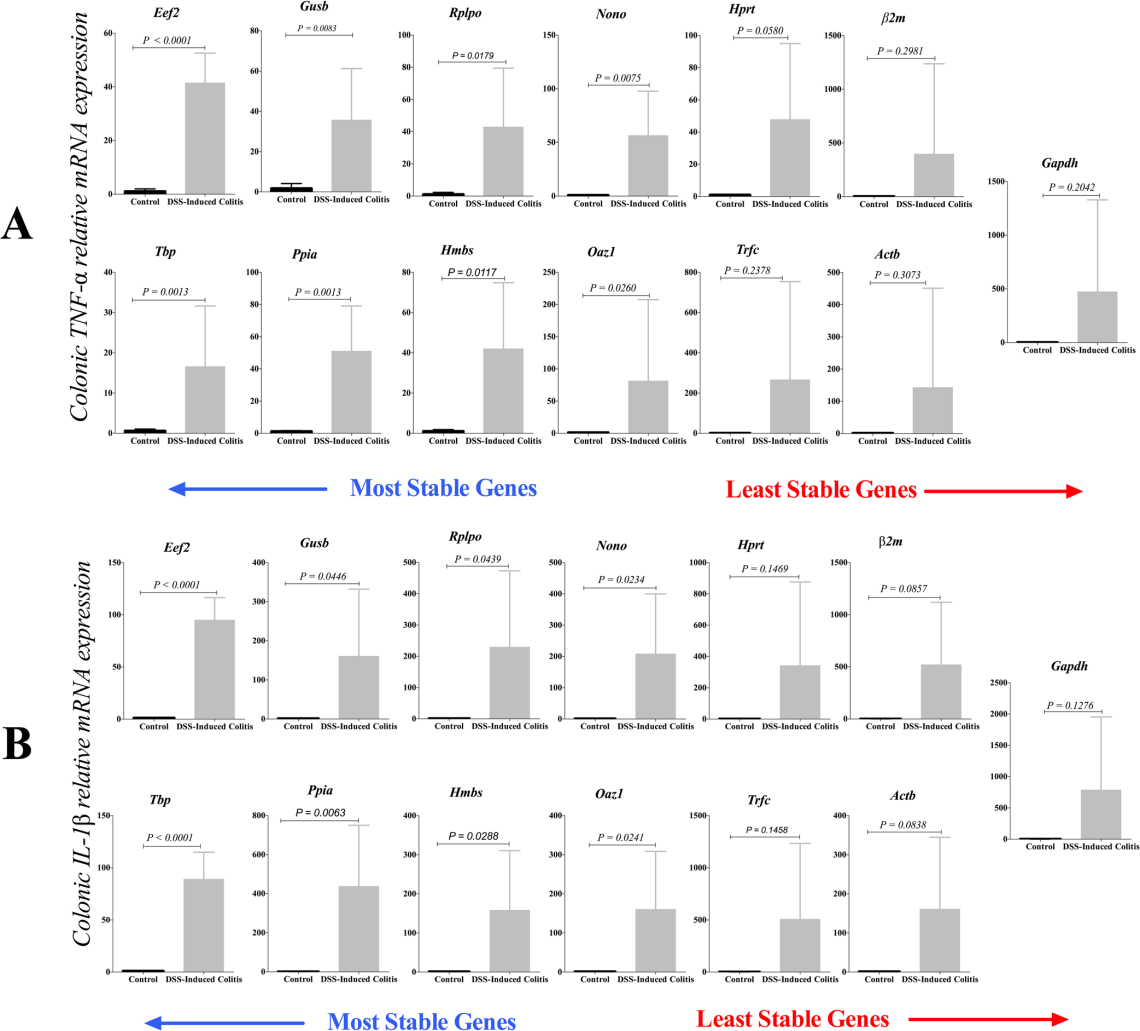
**Effect of reference gene selection on the relative expression of colonic TNF-α (A) and IL-1β (B).** Target gene expression was normalized against the 13 reference genes using comparative the ΔΔCt method. Significant differences between control and inflamed colon were seen only with the stable reference genes. Student’s t-test was used to compare the groups. Data is presented as the mean ± SD (n = 6/group).

The key effect of the 13 reference genes on quantifying relative mRNA expression of two target genes (*TNF-α* and *IL-1β*) in DSS-experimental colitis was investigated using comparative the ΔΔCt method (Fig 8). Our study indicates that reference gene selection significantly affects quantification of *TNF-α* and *IL-1β* mRNA expression levels, which can substantially change the results and their associated interpretation. When normalized against the sub-optimal or least stable genes, colonic *TNF-α* expression levels (Fig 8A) had a higher variability in DSS-experimental colitis, which shifted the results from a significant up-regulation to a non-significant up-regulation. The P value in the inflamed *vs*. control groups were for *Gapdh* (P = 0.2042), *Actb* (P = 0.3073), *β2m* (P = 0.2981), *Hprt* (P = 0.0580), *Trfc* (P = 0.2378); while the reference genes associated with a high stability profile demonstrated a significant *TNF-α* up-regulation in inflamed *vs*. control groups (*Eef2* P < 0.0022, *Tbp* P = 0.0013, *Gusb* P = 0.0083, *Ppia* P = 0.0013, *Rplp0* P = 0.0179, *Hmbs* P = 0.0117, *Nono* P = 0.0075, *Oaz1* P = 0.0260). The same pattern was demonstrated using *IL-1β* (Fig 8B). Using sub-optimal-reference genes, there was no difference in *IL-1β* mRNA levels between the inflamed and control groups (*Gapdh* P = 0.1276, *β2m* P = 0.1147, *Actb* P = 0.8308, *Trfc* P = 0.1458, *Hprt P = 0*.*1469*). Conversely, *IL-1β* relative mRNA expression levels were significantly up regulated in the inflamed colon when reference genes associated with a high stability profile were used (*Eef2* P < 0.0001, *Tbp* P < 0.0001, *Gusb* P = 0.0446, *Ppia* P = 0.0063, *Rplp0* P = 0.0043, *Hmbs* P = 0.0288, *Nono* P = 0.0234, *Oaz1* P = 0.0241). Additionally, the relative expression was calculated using using the difference in threshold cycle (ΔCt) method (S2 Fig).

### Reference genes stability in DSS-experimental colitis using External RNA Controls Consortium (ERCC)

**Fig 9:**
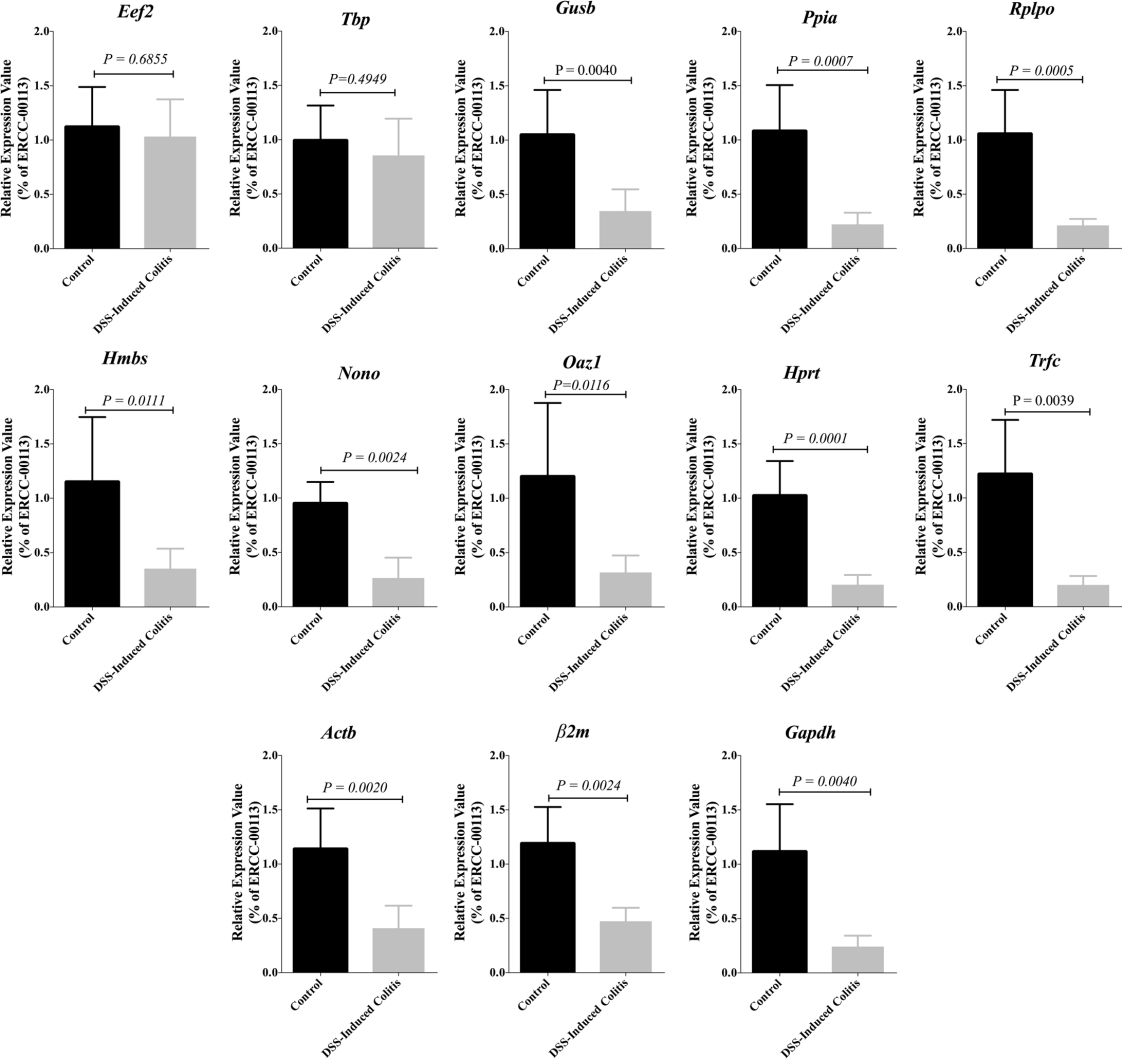
Changes in the expression levels of 13 selected reference genes in colon of control and DSS treated groups. The expression levels of these genes in colonic samples were normalized to the Ct values of ERCC-00113, an external control RNA. The graphs show relative expression values that calculated using the ΔΔCt method. Student’s t-test was used to compare the groups with significance level 0.05. Data are shown as mean ± S.D (n = 6 /group).

Moreover, to confirm our findings, the expression values of the 13 selected reference genes in control and DSS-groups were calculated based on the Ct values of control ERCC RNA (ERCC-00113). Although the expression levels of each of these genes in DSS group were down regulated when compared to the control group, variations in *Eef2* & *Tbp* expressions did not demonstrate any significant change (Fig 9). Colonic inflammation led to a more than 4-fold significant decline in the expression levels of 11 selected genes compared to control (*Gusb P = 0*.*0040*, *Ppia P = 0*.*0007*, *Rplp0 P = 0*.*0005*, *Hmbs P = 0*.*0111*, *Nono P = 0*.*0024*, *Oaz P = 0*.*0116*, *Hprt P = 0*.*0001*, *Trfc P = 0*.*0039*, *Actb P = 0*.*0020*, *β2m P = 0*.*0024*, *Gapdh P = 0*.*0040*), whereas the expression of *Eef2* (*P = 0*.*6855*) & *Tbp* (*P = 0*.*4949*) did not change significantly (Fig 9). Taken together, these data indicated that the expression of *Eef2 & Tbp* in colonic inflammation induced trough DSS administration is relatively stable compared to the 11 other reference genes.

## Discussion

To the best of our knowledge, this is the first systematic report on the stability of candidate gene mRNA expression used for data normalization in RT-qPCR experiments, which are used widely in the mouse DSS model. Normalization aims to remove sampling differences to identify actual gene-specific variations. For optimal reference genes, this variation should be minimal or absent. Therefore, the validation of suitable reference (endogenous control, housekeeping) genes is a critical step in mRNA expression analysis, because usage of an unstable or suboptimal gene could generate biased and ambiguous interpretations and conclusions.

In rodents, DSS is extensively used to induce experimental colitis and colitis-associated colonic cancer. With more than 4000 research articles, according to PubMed databases, DSS-induced colitis qualifies as a highly used model. Quantification of mRNA expression using RT-qPCR from these studies could provide a new mechanistic explanation in the context of colitis and assist in finding new therapeutic strategies. However, research groups worldwide use different reference genes when normalization is performed, and, thus, the contradictory results could be explained. In this study, 13 genes have been used to test their suitability, including the most broadly-used reference genes *Gapdh*, *Actb*, *Rplp0*, *β2m* and *Hprt*, which have been used in normalizing mRNA expression in normal intestinal mucosa, infected intestinal mucosa, intestinal carcinomas, colorectal cancer and different intestinal epithelial processes [23–25].

Here, we demonstrated the expression stability of the five most common reference genes, in addition to eight others that are used in DSS-experimental colitis, using different approaches including a final comprehensive ranking from the most stable to least stable reference gene expression. In this study, the expression of those widely used endogenous controls demonstrated a significant variability. In contrast, based on the predefined criteria as shown in results, we have identified two reference genes (*Tbp* and *Eef2*) that demonstrated optimal expression stability, which was not influenced by the experimental conditions (normal or inflammation). *Tbp* encodes a TATA-box-binding protein that is the common subunit essential for all three RNA polymerases to correctly initiate RNA transcription [26], while *Eef2* is an crucial factor for protein synthesis via promotion of the GTP-dependent translocation of the nascent protein chain from the A-site to the P-site of the ribosome [27]. *Tbp* and *Eef2* have recently been reported to be preferable to other commonly-used reference genes in various tissues. Kouadjo and colleagues [28] recognized *Eef2* as a constantly-expressed reference gene in various mouse tissues without significant variation in its expression, and it is not regulated by experimental conditions such as gonadectomy, steroid hormones and adrenalectomy. In the mouse intestine, Wang et al. [29] found that *Tbp* is an optimal reference gene for the normalization of gene expression and has stability suitable for a reference gene. Tatsumi et al. [30] reported that *Tbp* showed relative expression stability regardless of liver regeneration stages. Additionally, Svingen and colleagues [31] suggested that *Tbp* is an optimal reference gene for RT-qPCR experiments during early gonad differentiation in the mouse. This could be attributed to the fact that these two genes carry out their functions in the same way and capacity in different tissues and experimental conditions.

There are more than 700 published research articles, according to the PubMed database, that quantify gene expression in the DSS-model in mice, and all of them used *Gapdh* as reference gene in RT-qPCR expression analysis. Our results do not encourage the use of classical reference genes such as *Gapdh* for mRNA expression studies of the colonic mucosa. In particular, *Gapdh*, *Actb* and *β2m* exhibited high variability and the least stability in the presence of inflammation. These data corroborate previous studies showing extreme instability of these genes in different tissues or conditions. In IBD patients, intestinal inflammation significantly affects the stability of mucosal *Gapdh*, *Actb* and *β2m* expression, which displays high variability in healthy individuals and/or between the non-inflamed and inflamed mucosa [32]. Moreover, some studies reported fluctuations and variability in *Gapdh* expression in various tissues. Kouadjo and colleagues [28] reported that *Gapdh* and *Actb* are expressed in all mouse tissues, and there are significant differences in their expression levels between different tissues and between different experimental conditions. In the same context, Wang and colleagues [29] studied the stability of reference genes in the mouse small intestine and their results support our findings. They reported that expression of commonly used reference genes (*Gapdh*, *β2m*, *Aactb*) were relatively unstable. Moreover, *Gapdh* is regulated and showed high expression variability in mouse osteoblasts, osteoclasts and macrophages [33]. Adachi and colleagues [34] found that *Actb* and *Gapdh* exhibited expression instability and suboptimal performance as reference genes during retinal mouse development. The least stability of classic reference genes could be attributed to their functions, which could dysregulated under experimental or disease conditions and expression variations within different tissues. In the case of inflamed and non-inflamed mucosa, the suboptimal performance of these genes could result from the different cell populations in the mucosa. Therefore, depending on the relative proportion of different cell types that might have changed in the different samples included in the study (inflamed *vs*. control), this may explain the variability in expression. Findings from recent studies showed that in pure cell populations, *Gapdh* and *β2m* did not perform optimally as endogenous control genes [35]. *Gapdh* is a universal reference gene, but questions have begun to emerge regarding its validity as a reference gene. The previously mentioned publications, as examples, used different experimental models and human samples and reported that *Gapdh* is not a suitable reference gene, because of its high instability compared with other reference genes.

In general, our results show that the relative expression of a target gene may be miscalculated depending on the reference gene selected for correction, and this could shift the results from significant to non-significant, which will impact the final interpretation and conclusions, and cause potential errors in future research. This is especially true if fluctuations in reference gene expression stability have a significant effect on the relative targeted gene quantification. In our study, using the least stable reference genes for normalization resulted in no significant difference for *TNF-α* and *IL-1β* mRNA expression when the inflammatory state was compared to the control group. Conversely, the use of reference genes with high stability demonstrated a significant difference between the control and inflammatory groups. These findings are supported by previous studies inside and outside the gastrointestinal field, which report that normalization of a target gene using a sub-optimal or unstable reference gene induced fluctuations in the relative mRNA levels of the target gene and caused the final output to be non-significant. For gastrointestinal inflammation, Bamias and colleagues [32] validated reference gene stability in IBD patients and healthy individuals, and they concluded that using suboptimal reference genes such *Gapdh*, *Actb* and *β2m* to quantify mRNA expression such as for *TNF-α* in inflamed and non-inflamed colon and ileum altered the results from significant to non-significant. For other tissues and models, Ren et al. [36] tested the stability and suitability *Gapdh* and *Actb* in murine corneal disease models using target genes and they reported that *Gapdh* and *Actb* are suboptimal reference genes. Normalization of the target gene against either *Actb* or *Gapdh* resulted in conflicting relative expression ratios compared to the other tested reference genes. Also, circadian studies performed by Kosir and colleagues [37] investigated reference gene stability in different tissues and mouse strains, and they reported that *Gapdh* and *actb* showed the least stability. Selection of reference genes such as *Actb*, which is often used for analyses in individual mouse strains, may lead to an incorrect interpretation and could result in errors if used for normalization when different mouse strains are compared.

Since gene expression analysis involves a range of transcript abundances and differential expression ratios, it was important to use external RNA standards as quality controls for inter-run and cross-platform standardization to improve the accurateness of our class prediction based on the panel of biomarkers [38]. Recently, External RNA Controls Consortium (ERCC), an *ad hoc* group of 70 members from private, public and academic organizations led by the National Institute of Standards (NIST), has developed a test to use in gene expression applications [39, 40]. By using the ERCC as an external normalizer to investigate the expression levels of selected reference genes, we demonstrated that the inflammatory condition did not change the expression levels of optimal reference genes (*Tbp*, *Eef2*) but significantly down-regulated the suboptimal reference genes such *Gapdh*, *Actb and β2m*. This can be attributed to the fact that transcription rate of suboptimal reference genes can be influenced by cellular proliferation and functions such as glycolytic processes and protein phosphotransferase / kinase reactions. Therefore, in addition to the well-defined criteria to investigate the appropriateness of reference genes in RT-qPCR experiments, normalization to an ERCC RNA standard may be a useful confirmatory step for elucidating the stability of reference genes in the different experimental conditions.

Our findings are valid in, and refer to, colonic tissues isolated from C57BL/6 mice. This study reported the performance of 13 potential reference genes including the most common, widely used reference genes, but the potential that a more optimal reference gene combination may be found in the future cannot be excluded. Our findings only apply to the DSS-model in mice, and, therefore, do not eliminate the use of common reference genes such as *Gapdh*, *Actb* or *β2m* from being optimal reference genes in other experimental conditions, tissues or species.

## Conclusion

**Fig 10:**
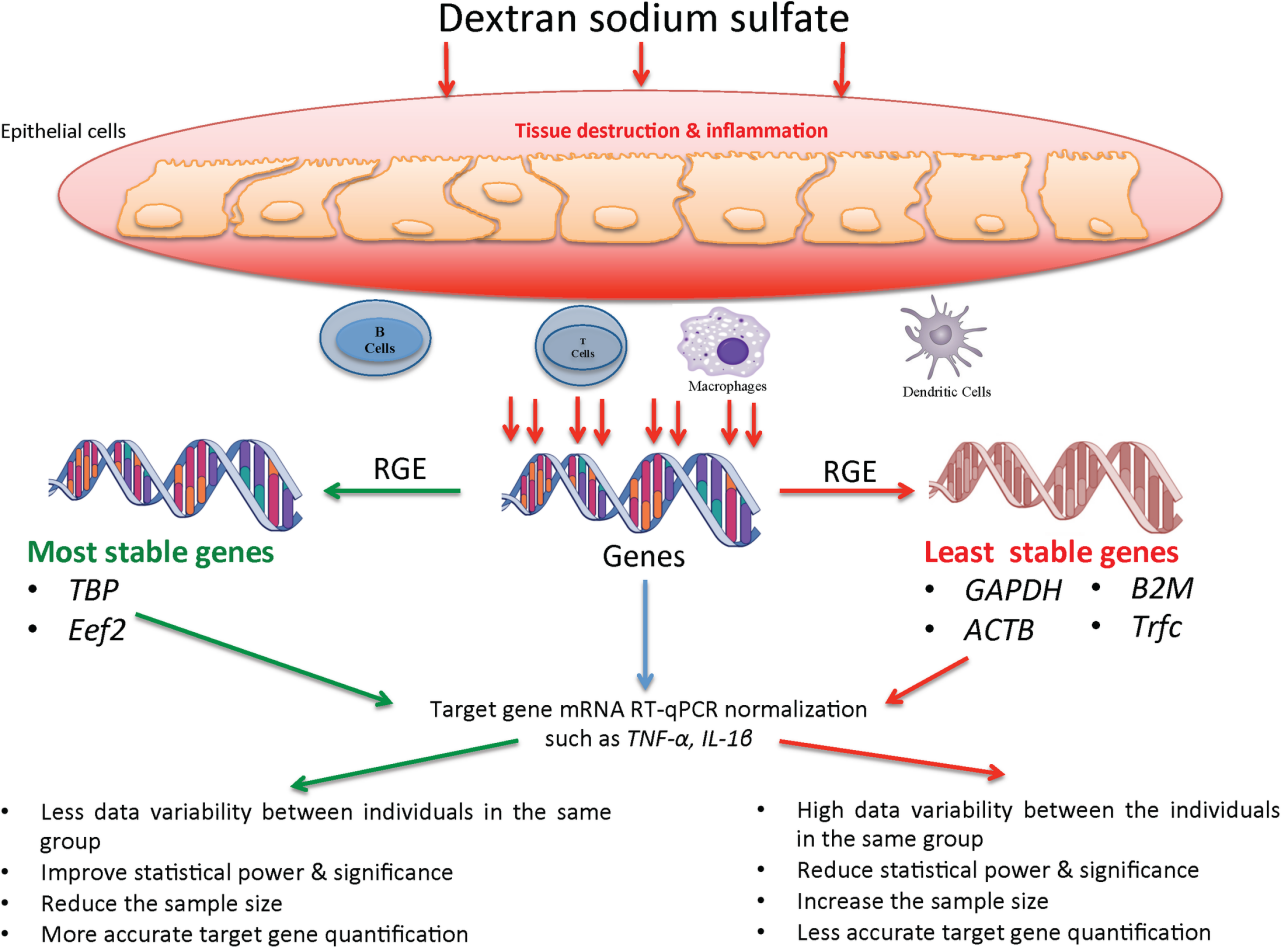
Illustration summary describes the effect of dextran sodium sulfate (DSS) on the reference genes expression (RGE) stability in the gastrointestinal mucosa and their impact when used to quantify mRNA level of target genes using RT-qPCR. Moreover, the immune cellular composition can greatly vary and can contribute to the variation of expression of a given gene. The least stable genes are the suboptimal genes that showed high variability (*Gapdh*, *Actb*, *β2m*, *Trfc*), while the most stable genes are the optimal genes that exhibited high stability (*Tbp*, *Eef2*).

In this study, 13 common reference genes were investigated using predefined criteria. Our comparative analysis of candidate reference genes recommended the use of *TBP/Eef2* as the appropriate reference genes for target gene normalization in C57BL/6 mice associated with the DSS-model. Suboptimal selection of reference genes may explain conflicting results for target gene expression in published studies using the mouse DSS-model because this study showed the instability and high variability of *Gapdh*, *Actb*, *Trfc* and *β2m* as reference genes (Fig 10). Most RT-qPCR results reported in the context of DSS-experimental colitis are still using these two traditional reference genes without pre-selection. Consequently, our study should facilitate the gene expression analysis in experimental models of DSS colon injury in mice. Because optimal reference gene normalization can improve statistical significance, power and dramatically reduce sample size optimization reference genes, it is a crucial step in mRNA quantification using real-time PCR. In any study that uses relative mRNA expression quantification, it is necessary to define a valid reference gene that takes into consideration the specific experimental conditions and the time course that is used, to avoid any misinterpretation of the results and to draw rigorous conclusions.

## Supporting Information

S1 Fig Amplification efficiency for the primers of candidate reference genes. The X-axis represents the log10 cDNA dilution series, and the Y-axis represents the cycle threshold (Ct). The primer efficiency (E) is calculated by [10^(1/-S)-1^] × 100%, where S represents the slope of the linear regression line. Roch Light Cycler Software was used for calculation.
(TIF)

S2 Fig **Effect of reference gene selection on the relative expression of colonic TNF-α (A) and IL-1β (B).** Target gene expression was normalized against the 13 reference genes using the difference in threshold cycle (ΔCt) calculation method. Significant differences between control and inflamed colon were seen only with the stable reference genes. Student’s t-test was used to compare the groups. Data is presented as the mean ± SD (n = 6/group).
(TIF)

## Abbreviations

Actb: β-actin
β2m: β2-microglobulin
CRP: C-reactive protein
CT: Threshold Cycle
DSS: Dextran Sodium Sulfate
Eef2: eukaryotic translation elongation factor 2
ERCC: External RNA Controls Consortium
Gapdh: glyceraldehyde-3-phosphate dehydrogenase
Gusb: Glucuronidase Beta
Hmbs: Hydroxymethylbilane synthase
Hprt: Hypoxanthine phosphoribosyl transferase
IBD: Inflammatory bowel disease
IL-1β: Interleukin*-1 β*
IL-6: Interleukin-6
MPO: Myeloperoxidase Activity
Nono: Non-POU Domain Containing, Octamer-Binding
Oaz1: Ornithine Decarboxylase Antizyme 1
Ppia: Peptidylprolyl Isomerase A
RGE: Reference genes expression
Rplp0: Ribosomal Protein Large P0
RT-qPCR: quantitative real time PCR
SD: Standard Deviation
Tbp: TATA-box-binding protein
Tfrc: Transferrin Receptor
TNF-α: Tumor necrosis factor-α
UC: Ulcerative colitis
