# Supplementary figures and images for: Stability of Reference Genes for Messenger RNA Quantification by Real-Time PCR in Mouse Dextran Sodium Sulfate Experimental Colitis
Source: PLoS One. 2016 May 31;11(5):e0156289. doi: 10.1371/journal.pone.0156289 (PMC4886971; doi:10.1371/journal.pone.0156289)

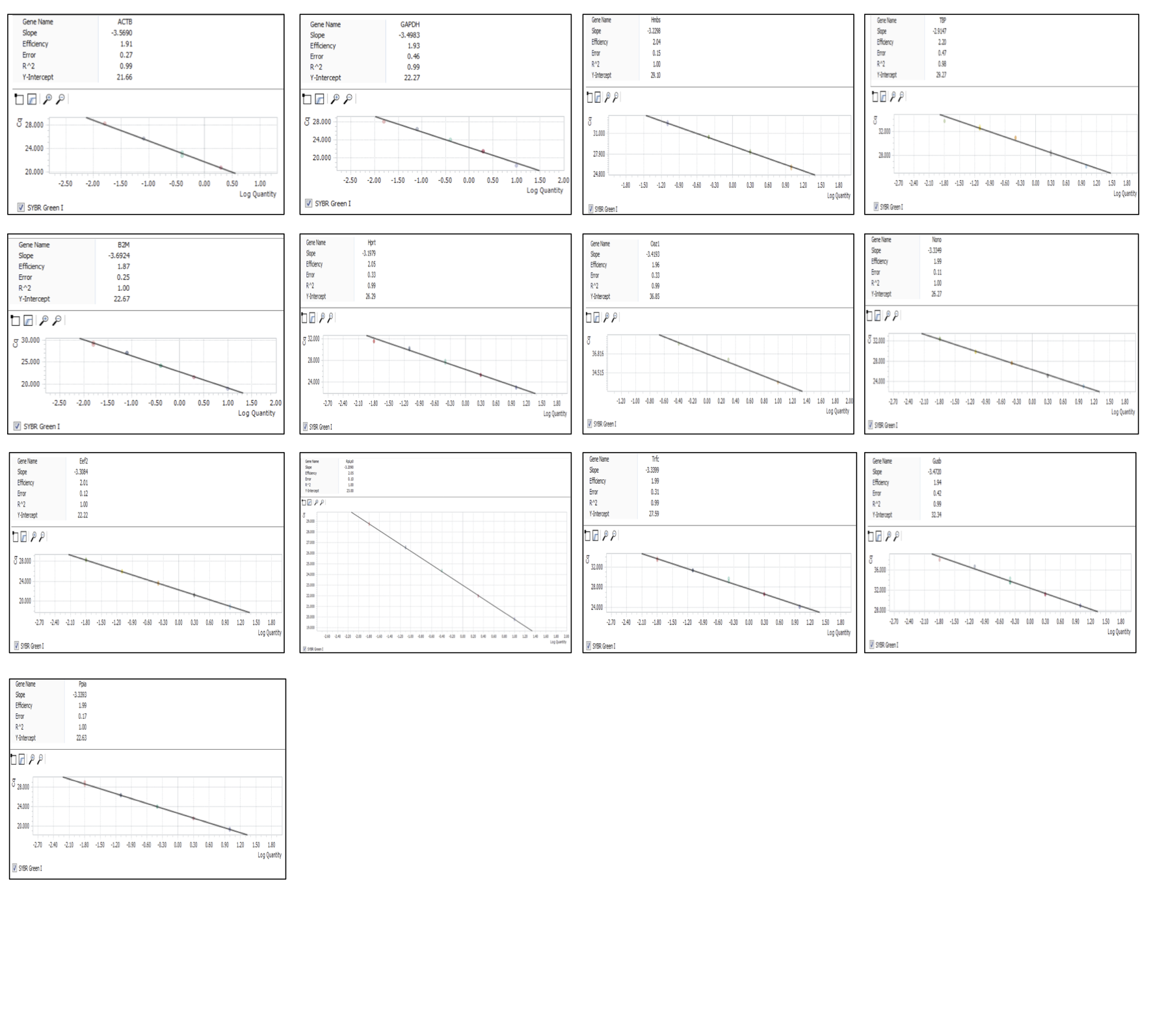

Supplement: S1 Fig — The X-axis represents the log10 cDNA dilution series, and the Y-axis represents the cycle threshold (Ct). The primer efficiency (E) is calculated by [10(1/-S)-1] × 100%, where S represents the slope of the linear regression line. Roch Light Cycler Software was used for calculation. (TIF) [file pone.0156289.s001.tif]

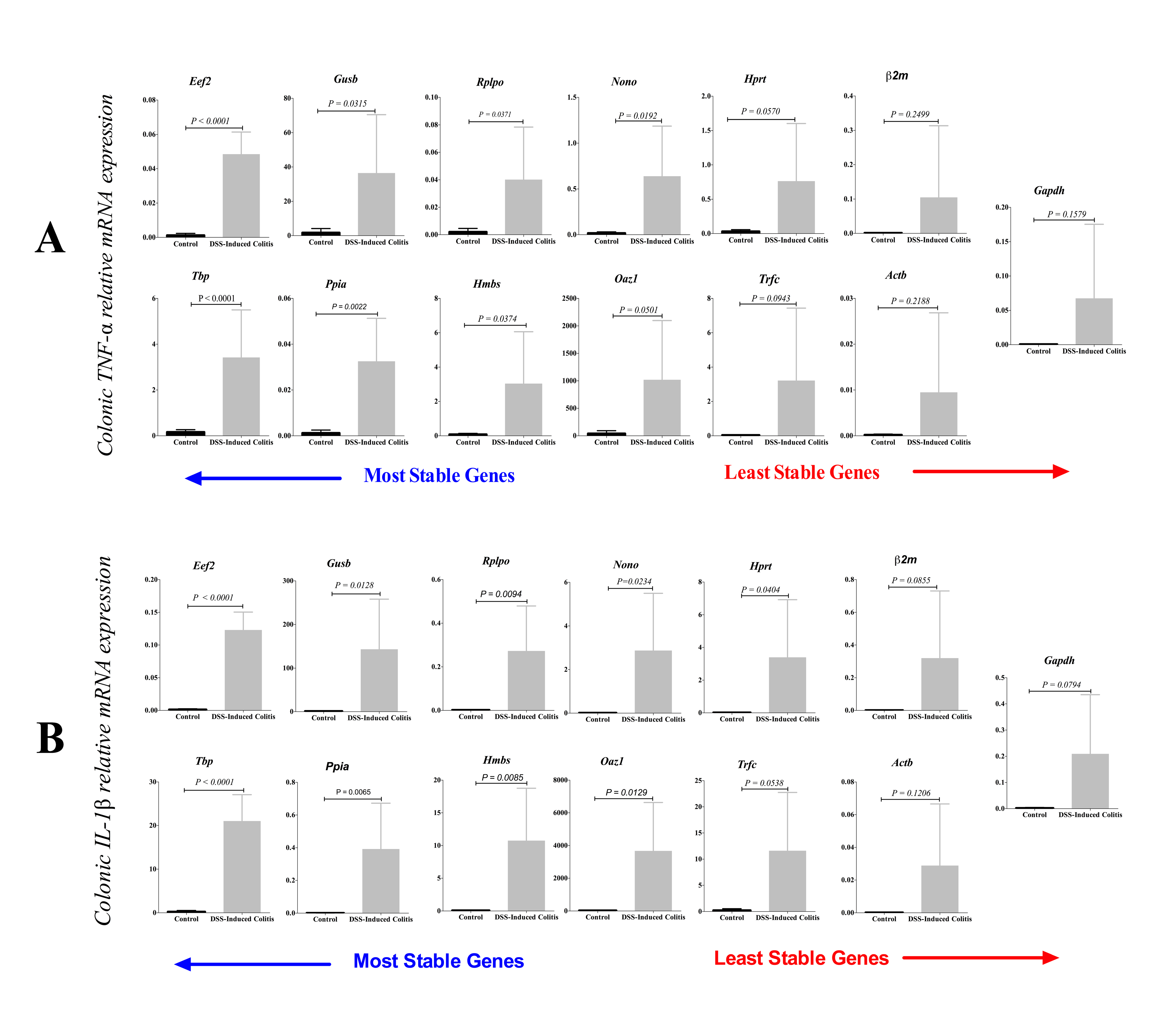

Supplement: S2 Fig — Effect of reference gene selection on the relative expression of colonic TNF-α (A) and IL-1β (B). Target gene expression was normalized against the 13 reference genes using the difference in threshold cycle (ΔCt) calculation method. Significant differences between control and inflamed colon were seen only with the stable reference genes. Student’s t-test was used to compare the groups. Data is presented as the mean ± SD (n = 6/group). (TIF) [file pone.0156289.s002.tif]
